# Validity and Reliability of the 30-s Continuous Jump for Anaerobic Power and Capacity Assessment in Combat Sport

**DOI:** 10.3389/fphys.2018.00543

**Published:** 2018-05-15

**Authors:** Drazen Čular, Vladimir Ivančev, Alessandro M. Zagatto, Mirjana Milić, Tea Beslija, Maha Sellami, Johnny Padulo

**Affiliations:** ^1^Faculty of kinesiology, University of Split, Split, Croatia; ^2^Croatian Institute for Kinesiology and Sport, Split, Croatia; ^3^Faculty of Sciences, Department of Physical Education, UNESP–São Paulo State University, Bauru, Brazil; ^4^University eCampus, Novedrate, Italy

**Keywords:** children, metabolic demand, testing, kicking combat sport, phosphagen pathway

## Abstract

Cycling test such Wingate anaerobic test (WAnT) is used to measure anaerobic power (AP), but not anaerobic capacity (AC, i.e., the metabolic energy demand). However, in sports that do not involve cycling movements (Karate), the continuous jump for 30 s (vertical jumps for 30 s) has been extensively used to measure anaerobic performance in all young athletes. Limited information’s are available concerning its validity and reliability especially in children. As such, the current study aimed to test validity and reliability of a continuous jumps test (the CJ30s), using WAnT as a reference. Thirteen female Karate kids (age: 11.07 ± 1.32 years; mass: 41.76 ± 15.32 kg; height: 152 ± 11.52 cm; training experience: 4.38 ± 2.14 years) were tested on three separate sessions. The first and second sessions were used to assess the reliability using Intra-class correlation coefficient (ICC) of CJ30s, whereas on the third session WAnT was administered. Following CJ30s and WAnT, we assessed AP (1/CJ30s, as jump height [JH], fatigue index [FI], and blood lactate [BL]; 2/WAnT, as mechanical power [P], FI, and BL) and AC as the excess post-exercise oxygen consumption (EPOC). Large/highly significant correlations were found between CJ30s and WAnT EPOCs (*r* = 0.730, *P* = 0.003), and BLs (*r* = 0.713, *P* = 0.009). Moderate/significant correlations were found between CJ30s and WAnT FIs (*r* = 0.640, *P* = 0.014), CJ30s first four jumps mean JH and WAnT peak P (*r* = 0.572, *P* = 0.032), and CJ30s mean JH and WAnT mean P (*r* = 0.589, *P* = 0.021). CJ30s showed excellent and moderate reliability (ICC) for AP (maximal JH 0.884, mean JH 0.742, FI 0.657, BL 0.653) and AC (EPOC 0.788), respectively. Correlations observed especially in terms of AC between CJ30s and WAnT provide evidence that former may adequately assess anaerobic performance for the young combat athlete. CJ30 is a reliable test and allow an easy assessment of AP and AC in karate children.

## Introduction

The anaerobic capacity (AC) is defined as the maximal amount of energy that can be generated over a given period of time using anaerobic sources of energy (i.e., phosphagen and glycolytic energy pathways). It has been well demonstrated that AC depends on exercise type, involved muscle group (Bangsbo et al., 1990). Both anaerobic power (AP) and AC must be excellent allowing competitors to repeat high intensity bouts of activity with minimal rest period such in combat sports (Mendez-Villanueva et al., 2012). In these contact sports, high fitness level requires complex skills and tactical excellence for success (Ghrairi et al., 2014), however, one very important component to success is to perform powerful and fast blows, which depend significantly of AP and AC (Heller et al., 1998; Hubner-Wozniak et al., 2011).

Martial arts (e.g., karate, taekwondo, kickboxing) have specific psychophysiological demands (Alesi et al., 2014; Padulo et al., 2014a) and require well-developed muscle power in both the upper and lower limbs (Tabben et al., 2013). These sport characteristics demonstrate the importance of anaerobic fitness development via AP and AC to achieve high fitness level. Among these aspects of fitness, muscle strength of the lower limbs is particularly important because it is crucial for kicking. To measure the anaerobic metabolism in lower limbs, researchers of the Wingate institution developed the Wingate test (WAnT) in cycle ergometer as a method of measuring maximal anaerobic power (peak and mean), as well as anaerobic fatigue (Bar-Or, 1987). However, procedures of WAnT evaluate the mechanical outcomes (i.e., mechanical power, jumping performance) representing an estimation of anaerobic power instead of measurements of metabolic variables that represents the anaerobic capacity (Minahan et al., 2007).

Recently, several studies have reported the possibility to estimate separately the oxygen equivalents from phosphagen and glycolytic energetic pathways during running and cycling using the fast component of excess post-exercise oxygen consumption (EPOC-fast) and net blood lactate accumulation (Δ_[La]_), respectively (Zagatto et al., 2016a). Bertuzzi et al. (2010) and Miyagi et al. (2017), reported that the sum of both correspond to the AC predicted level. This procedure may help to estimate separately each metabolism and its relevance using specific exercise (Zagatto et al., 2016b). This method could be also used to estimate the anaerobic capacity in combat sports such karate in association with short and briefs efforts such as jumping. As Jumping has been a staple for combat athletes for a long, long time, it is essential to adopt a jumping test that allow exploration of anaerobic performance in these sports and for all age categories (Dal Pupo et al., 2014). For Sands et al. (2004), comparison between the 60-s Bosco test and WAnT showed different aspects of anaerobic performance and reported that the jump test is more suitable for athletes who are familiarized with it (i.e., Karate). But, more recent, Theodorou et al. (2013) found that a modified 30 s Bosco vertical jump test is a valid tool compared to WAnT with significant correlation between absolute jump high and absolute power measured during WAnT in young moderately trained athletes. It is important to mention that efforts to develop and exploit valid testing design and procedures are seriously hampered by a significant and continuing lack of basic scientific knowledge concerning the AC and AP in combat sport such as karate and especially in children (Melhim, 2001; Doria et al., 2009; Driss and Vandewalle, 2013; Obminski et al., 2013; Herrera Valenzuela et al., 2014).

To the best of our knowledge, some studies have demonstrated a strong relationship between cycling and jumping tests despite a low to moderate sample size (Hoffman et al., 2000; Driss and Vandewalle, 2013; Dal Pupo et al., 2014). For example, Dal Pupo et al. (2014), investigated the test–retest reliability and concurrent validity of the 30 s continuous jump (CJ30) test using the WAnT in young (23 years old) male volleyball players. They found strong correlations between the mean height of the first four jumps of CJ30s and WAnT. They concluded that the CJ30s would be a reliable test to measure anaerobic performance in young athletes (∼20 years old) and could replace the WAnT.

As mentioned, most studies investigated correlation between jump test and WAnT in young population (De Siati et al., 2016; Laffaye et al., 2016; Nikolaidis et al., 2016a,c), while few studies have explored the effect of age on this relationship in terms of anaerobic performance. In fact, immaturity of anaerobic metabolism is a main determinant factor that could influence the results of tests and therefore relationship between different aspect of anaerobic performance (Van and Dore, 2002). As there are several possible reasons for a lower glycolytic activity in children compared with adult (Van and Dore, 2002). In this context, Nikolaidis et al. (2016c) investigated the effect of age on the relationship between jumping and cycling tests in handball players. They observed correlation in muscle power and heart rate level between Bosco test and WAnT for all age groups (12–15, 15–18, 18–25, and 25–35 years). In addition, the influence of the Bosco test and WAnT on muscle power varied, especially in the younger age (≤16 years) group in Volley-ball sport (Nikolaidis et al., 2016a). As such, the aim of this study was to determine the test–retest reliability and concurrent validity of the continuous jump test performed over 30 s (CJ30s) for anaerobic capacity evaluation of Karate children (∼11 years), using WAnT as a reference tool. To the best of our knowledge, this study is the first attempt to determine the anaerobic capacity and power by jump test due to increased activity of lower limbs during the fight; then, we aimed to improve the ecological validity compared to cycling Wingate test.

## Materials and Methods

### Participants

Thirteen children female karateka (Age: 11.07 ± 1.32 years; body weight: 41.76 ± 15.32 kg; body height: 152 ± 11.52 cm; training experience: 4.38 ± 2.14 years) volunteered to participate in this study. The parents reviewed and signed consent forms approved by the local Ethics Committee for Human Research (ECHR) of the University of Split: “Ethical Committee of the Faculty of Kinesiology (ECFK).” The “Ethical Committee of the Faculty of Kinesiology” approved the entire study design which has been conducted according to the principles expressed in the Declaration of Helsinki. Participants trained on a regular basis (three sessions per week) with 2′ of jumps before the training) during 2 years and they were currently competing at the national level. Inclusion criteria included the absence of the following: contraindications to maximal exercise testing (e.g., cardiovascular or pulmonary disease); metabolic syndrome symptoms (e.g., hypertension, impaired fasting glucose), joint and muscle injuries.

### Experimental Procedures

Participant were tested in three separate sessions with an interval of 48-h between each session. The first and second sessions were used to determine the reliability of the Continuous Jump test (CJ30s), while the third session was used to perform Wingate Anaerobic Test (WAnT) that was considered as standard test to investigate concurrent validity.

Before anaerobic exercises, participants were familiarized with testing procedures to negate learning effect. During familiarized sessions, participants were asked to perform the CJ30s and the sprint cycling test WAnT under same condition (clothing, time of date, warm-up).

Both exercises (CJ30s and WAnT) were separated by 72 h of rest to allow complete recovery. For CJ30s, two sessions were sufficient to learn correct jumping technique and to familiarize with basic takeoff and landing position. Similarly, for WAnT, all participants performed the best exercise technique after two sessions. Participants performed all sessions during the morning (between 8:00 and 10:00 am).

Anthropometric measurements were performed during the first session. Participants avoided physical activity during the 48 h preceding each test. They were asked to abstain from high glycemic loads, saturated and trans-fatty acids, caffeine, alcohol, drugs, vitamins or supplements, and low-fiber diets for the duration of the study. All tests were performed in the morning 2 h postprandial and under same condition (temperature: ∼24°C inside laboratory) to avoid any circadian effect (Ammar et al., 2015). During the tests the participants remained 10-min sitting in a chair to measure the resting blood lactate value (baseline), as well as after the tests the same procedures was adopted (i.e., 10-min of resting) to measure the blood lactate response and mainly the fast component of EPOC-fast. Blood samples were collected at rest and after both tests (1st, 3rd, and 5th min of recovery). In addition, energetic contributions from phosphagen (EPCr) and glycolysis (E_[La]_) were estimated during both tests (CJ30s and WAnT).

### CJ30s Testing

The CJ30s was preceded by a general warm-up (<50% VO_2max_) composed of jogging, walking, and stretching for about 15 min (Tomaras and MacIntosh, 2011; Sellami et al., 2017a,b) and specific warm-up composed of five joint mobility exercises (one set of 10-s) with emphasis on the lower limbs and 6′ walking on treadmill (0% at 4.5 km/h); 2′ of recovery and two jumps with 1-min in-between to avoid any fatigue effects (Chamari et al., 2001). The test started (∼3′) when the oxygen consumption and heart rate reached about the baseline value (compared to the rest before the warm-up) (Chamari et al., 2001).

The CJ30s consisted of maximal continuous vertical jumps performed for 30 s in according to Dal Pupo et al. (2014). Participants were required to keep the trunk as vertical as possible, and hands were placed on hips. According to recommendations of the protocol, participant was also asked to flex their knees at ∼90° in the transition between negative/positive phases (Padulo et al., 2013), which is considered the best angular position to maximize the vertical jump performance (Gheller et al., 2015). To better replicate each jump (i.e., braking phase corresponding at knee 90°, ∼5° as tolerance (Gheller et al., 2015); previously standardized 90° with goniometer/accelerometer) an electronic “Bip” audio feedback (SpinGNSSv2, SpinItalia, Rome) via computer was used when each participant reached the knee at 90°. The electronic audio feedback (Vando et al., 2014) includes a customized accelerometer with sample rate 100-Hz (SpinGNSSv2, SpinItalia, Rome), secured by elastic band (Wetrap) on the rectus femoris (in the middle), connected via Bluetooth to the Notebook and managed from Bridge software (LagalaColli_Bridge V. 8.4.14.5). Verbal feedback will be provided to the subject during the test to encourage them to maintain maximum performance (i.e., explosive continuous jump) until the end of the test. All jumps was assessed with Optojump Next (Microgate, Italy) (Attia et al., 2017) and the best performance was then used to compare with WAnT performance.

The maximal jump height (*H*_MAX_), the mean jump height of the first four jumps (*H*_MEAN_4J_), the mean jump height of all jumps (*H*_MEAN_), the number of jumps (as total jumps) and the fatigue index were calculated. The fatigue index was obtained considering the first (*H*_MEAN_4J_) and the last (*H*_MEAN_end4J_) four jumps of the test (Maud and Foster, 2009), according to Eq:

$$\begin{array}{r} Fatigue\;Index\;=\;[(H_{MEAN\_4}-H_{MEAN\_end4J})/H_{MEAN\_4J}]\\ \times \;100 \end{array}$$

The *H*_MEAN_4J_ was used as indicator of peak power in an attempt to determine an analogous measure in the CJ30s. This is similar to WAnT and is generally obtained during the first 5 s of the test.

### Wingate Anaerobic Test (WAnT)

Wingate anaerobic test was performed with a specific cycle-ergometer (Monarch, Peak Bike 894e, MONARK, Sweden), according to the protocol used by Inbar et al. (1996). Participants were adjusted on cycle ergometer (the seat higher in relationship of the length leg and the distance of the handlebar (Padulo et al., 2012, 2014b, 2015, 2016).

Before the start of WAnT, participants performed a general warm-up (<50% VO_2max_) composed of jogging, walking, and stretching for about 15 min (Tomaras and MacIntosh, 2011; Sellami et al., 2017a,b) followed by specific cycling exercise (5-min in the cycle ergometer with a load of 35 W). The test started 2-min after the warm-up. WAnT was performed at maximal intensity for 30-s with a load corresponding to 7.5% of body mass (previously calculated). Resistance was applied after 3-s and the revolution per minute reached almost 70 of maximal acceleration with no load. Participants were instructed to remain seated throughout the test and received verbal encouragement to sustain their maximum effort throughout the test. A one-minute period of cycling with no load was included at the end of the test. The following variables were obtained in WAnT with the Monark Software (Monark ATS Software, MONARK, Sweden): Peak power, mean power, lowest power and fatigue index (Maud and Foster, 2009) calculated according to Eq:

$$\begin{array}{r} Fatigue\;Index\;=\;[(Peak\;power\;-\;lowest\;power)/Peak\;power]\\ \times \;100 \end{array}$$

### Physiological and Metabolic Measurements

Arterialized blood samples (20 μL) were collected from the earlobe at rest and after (1st, 3rd, and 5th min of recovery) WAnT and CJ30s, respectively. Lactate concentration was determined through a portable lactate scout Lactate Pro2 (ARKRAY, Inc., Kyoto, Japan), which was calibrated before each measurement according to the manufacturer’s manual. The highest blood lactate concentration measured after the test was assumed as peak value and the net blood lactate concentration (Δ_[La]_) was determined by the difference between peak and baseline values.

**FIGURE 1:**
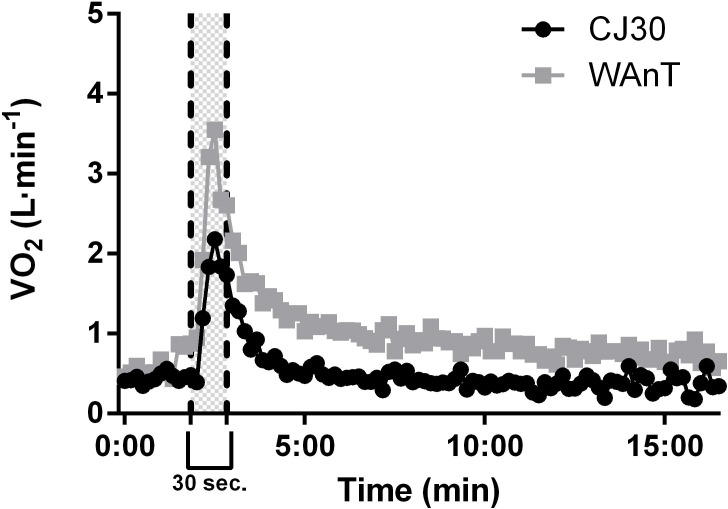
Oxygen uptake pre, during and post a Wingate anaerobic test (WanT) and CJ30s (30 s continuous jumps test).

The heart rate (Garmin^TM^), the oxygen uptake (VO_2_) and carbon dioxide production were measured and recorded breath-by-breath (**Figure 1**) using a metabolimeter system (K5, Cosmed, Italy) for the duration of the test (i.e., 10′ at baseline – warm-up – 30 s exercise – 15 min at rest). Before each test, the gas analyzer was calibrated using a high-precision gas mixture (5.06% CO_2_ and 16.02% O_2_) and the spirometer with a 3-liter syringe (Hans Rudolf, Kansas City, MO, United States), in accordance with the manufacturer’s instructions. During the calibration process the gas analyzer was fixed on a pedestal to avoid any influence of the external load (i.e., metabolimeter’s weight) during the jumps or pedaling; while the Omnia software (Cosmed, Italy) was able to discriminate with markers each phase. Energetic contributions from phosphagen (E_PCr_) and glycolysis (E_[La]_) were estimated during both tests (CJ30s and WAnT). The E_PCr_ contribution was considered as the EPOC-fast, which was estimated by multiplication of the amplitude and the time constant of the fast component of a bi-exponential model, while the E_[La]_ energy was estimated by Δ_[La]_, considering a value of 1 mmol⋅L^-1^ to be equivalent to 3 mL O_2_/kg body mass (di Prampero and Ferretti, 1999). Both energetic pathways were calculated using the the GEDAE-LaB software by Bertuzzi et al. (2016). Finally, the anaerobic capacity corresponded sum of both E_PCr_ and E_[La]_.

### Statistical Analysis

The data are expressed as mean and standard deviation (SD). Shapiro–Wilk Test was used to verify normal distribution of the data. The test–retest reliability was determined by calculating the Intra-class Correlation Coefficient (ICC). The ICC values were classified as follows: <0.4 = poor reliability; 0.4–0.75 = fair to good reliability; and >0.75 = excellent reliability (Fleiss, 1991). Anyway for the same variables we calculated the coefficient variation (CV) from duplicate measurement using Root Mean Square method (Hyslop and White, 2009). The typical error measurement (as Standard Error) was calculated with a linear regression analysis. The effect size was calculated as Cohen’s (1988). A paired *t*-test was used to shown the level significance between test–rest data. Pearson’s correlation coefficients were used to establish the correlation between WAnT and CJ30s parameters. Considering the strong reliability of test–retest previously analyzed, the best data were used to compare with WAnT parameters. Bland–Altman plot were used to verify the measurement agreement for anaerobic contribution and delta lactate between WanT and CJ30s, respectively (Bland and Altman, 1986). The following criteria were adopted for interpreting the magnitude of correlation between variables: <0.1, trivial; 0.11–0.3, small; 0.31–0.5, moderate; 0.51–0.7, large; 0.71–0.9, very large; and 0.91–1.0, almost perfect (Hopkins et al., 2009). The analyses were performed with the Statistical Package for Social Sciences (SPSS Inc., v.17.0, Chicago, IL, United States) and MedCalc^®^ (v.11, United States) and the level of significance was fixed with *P* < 0.05.

## Results

**FIGURE 2:**
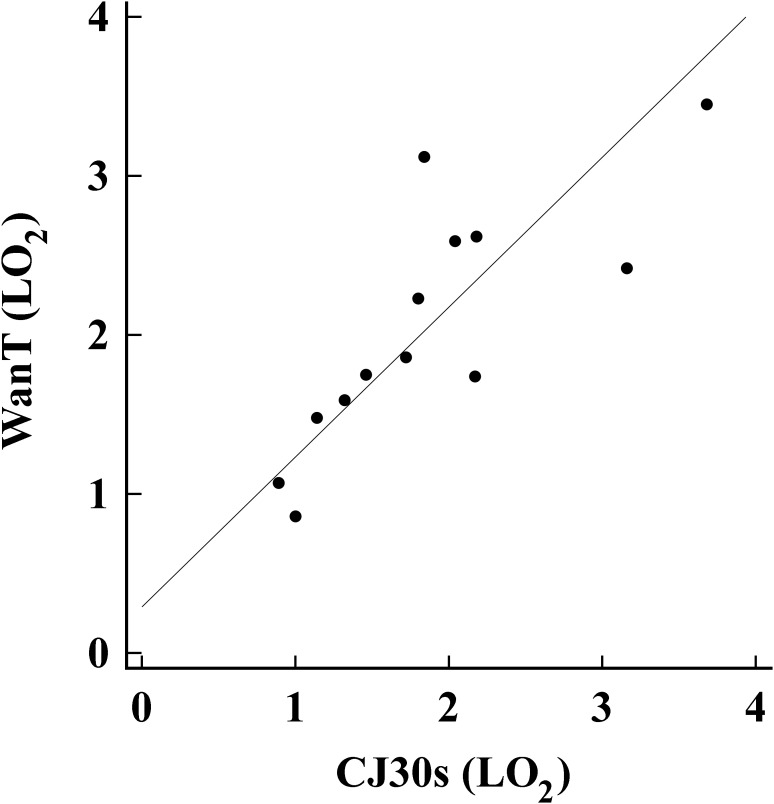
Anaerobic Capacity (LO2) relationship between the WanT and CJ30s.

The analysis showed that normal distribution can be accepted for both the variables CJ30s (*W* = 0.952, *P* = 0.594) and WAnT (*W* = 0.978, *P* = 0.964). **Table 1** shows the test-retest values and the differences of the CJ30s. These results demonstrated that both mechanical variables (jump performance) as well as anaerobic capacities were reliable. While the WAnT outcomes are presented in **Table 2**. For both test (WAnT/CJ30s) no significantly differences showed for blood lactate at baseline conditions (1.30 ± 0.55/1.26 ± 0.36 mmol⋅L^-1^ with *P* = 0.80). No significance differences was found between both test (WAnT/CJ30s) for Anaerobic capacity, E_PCr_, E_[La]_ with *P* = 0.11, *P* = 0.14, *P* = 0.25, respectively. Large correlations were found between anaerobic capacity of CJ30s and WAnT (*r* = 0.730, *P* = 0.003; **Figure 2**), the blood lactate between CJ30s and WAnT found (*r* = 0.713, *P* = 0.009), demonstrating the concurrent validity. In addition, moderate correlations of fatigue index between CJ30s and WAnT (*r* = 0.640, *P* = 0.014), the mean height of the first four jumps of CJ30s and the WAnT’s peak power (*r* = 0.572, *P* = 0.032), the mean vertical jump height of CJ30s and the mean power of WAnT (*r* = 0.589, *P* = 0.021) were found.

**Table 1:** Reliability and comparison of the continuous jump measures.

Variable	Mean ± SD		CV	SE	*t*-test	ES	ICC	CI (95%)
	Test	Retest						
*H*_MAX_ (cm)	18.58 ± 2.64	18.74 ± 2.44	4.77	1.217	0.656	0.054	0.884	0.662–0.965
*H*_MEAN_ (cm)	13.63 ± 1.63	13.79 ± 1.55	5.97	1.812	0.646	0.83	0.742	0.341–0.917
Total Jumps (*n*)	35.75 ± 3.39	35.17 ± 3.13	6.04	2.960	0.526	0.178	0.568	0.047–0.851
FI (%)	31.82 ± 17.10	32.48 ± 12.02	26.57	8.985	0.630	0.039	0.657	0.186–0.886
[La]_peak_ (mmol⋅L^-1^)	5.76 ± 2.03	6.02 ± 1.58	26.06	1.504	0.651	0.113	0.522	0.033–0.809
Δ_[La]_ (mmol⋅L^-1^)	4.18 ± 1.66	4.66 ± 1.64	36.21	1.662	0.428	0.235	0.653	-0.092–0.893
AC (LO_2_)	1.79 ± 0.91	1.81 ± .085	32.33	0.561	0.927	0.015	0.783	0.474–0.926
E_PCr_ (LO_2_)	1.15 ± 0.48	1.10 ± 0.41	33.93	0.447	0.800	0.025	0.588	0.128–0.840
E_[La]_ (LO_2_)	0.63 ± 0.44	0.67 ± 0.46	37.65	0.293	0.397	0.078	0.828	0.565–0.939

*Value expressed as Mean and SD (Standard Deviation) for H_MAX_, maximal jump height; H_MEAN_, mean height considering all jumps; Total Jumps (within 30 s); FI, fatigue index; La_peak_ and Δ La_peak_, blood lactate peak; AC, anaerobic capacity; E_PCr_, anaerobic alactic; E_[La]_, anaerobic lactic; CV, coefficient variation; SE, standard error; paired t-test, P-value; ES, effect size; ICC, intra-class correlation coefficient; CI, confidence interval.*

**Table 2:** Wingate anaerobic test (WAnT) measurement for all participants.

Variable	Mean ± SD
Peak power (W⋅kg^-1^)	5.41 ± 1.26
Mean power (W⋅kg^-1^)	3.92 ± 0.95
FI (%)	57.70 ± 13.52
[La]_peak_ (mmol⋅L^-1^)	6.77 ± 2.25
AC (LO_2_)	2.05 ± 0.74
E_PCr_ (LO_2_)	1.33 ± 0.40
E_[La]_ (LO_2_)	0.72 ± 0.43

*Value expressed as Mean and SD (Standard Deviation); FI, fatigue index; La_peak_, blood lactate peak; AC, anaerobic capacity; E_PCr_ = anaerobic alactic; E_[La]_, Anaerobic Lactic.*

**FIGURE 3:**
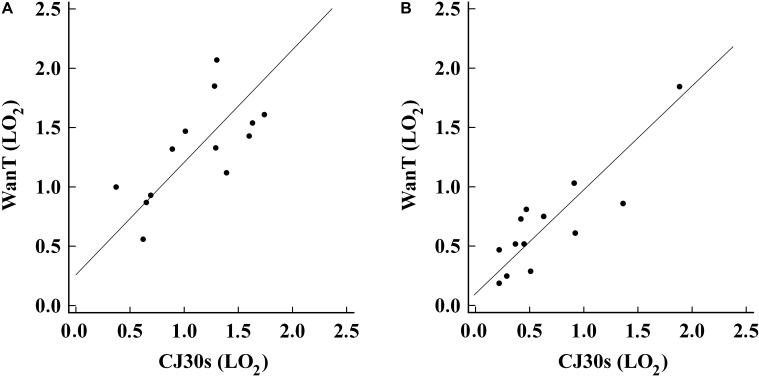
Anaerobic Capacity (LO2) relationship between (E_PCr_ = **A** and E_[La]_ = **B**) the WanT and CJ30s.

**FIGURE 4:**
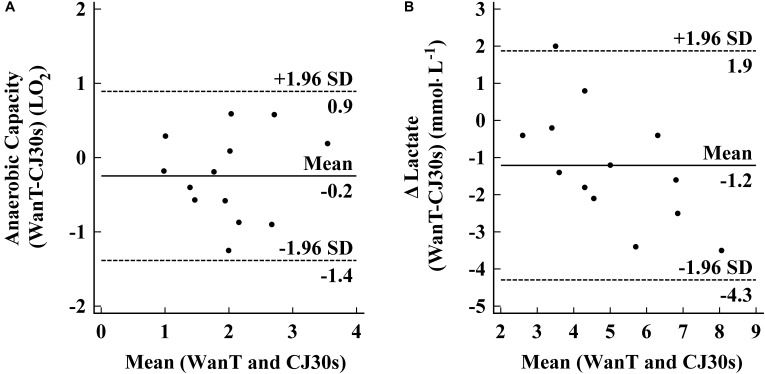
Bland–Altman plots showing the agreement between WanT and CJ30s sessions for **(A)** Anaerobic capacity and **(B)** Δ Blood lactate (difference between peak and baseline values).

The E_PCr_ measured during CJ30_S_ and WAnT was significantly correlated (*r* = 0.645 with *P* = 0.0127), as well as the E_[La]_ (*r* = 0.807 with *P* = 0.0005), respectively (**Figure 3**). The Bland–Altman test showed a mean = -0.25 and a 95% limits of agreement = ± 1.16 for the anaerobic capacity measurements, whereas a mean = -1.21 and a 95% limits of agreement = ± 3.15 for the lactate measurements, as showed in **Figure 4**.

## Discussion

The main findings of the current study were the validation of reliability of anaerobic capacity measured during CJ30s and the significant and large correlation between anaerobic capacity estimated during CJ30s and WAnT reporting the concurrent validity. In addition, we found interesting significant correlation between E_PCR_ and E_[La]_ measured during both tests. Moreover, we found moderate and significant correlations between CJ30s and WAnT for mechanical outcomes in Karate children.

It is well demonstrated that prepubertal children have reduced activity of phosphofructokinase-1 and lactate dehydrogenase enzymes markedly observed during intense efforts (Ratel et al., 2002). In fact, Hebestreit et al. (1996) found that during a maximal 30-s all-out effort the blood lactate concentration was 5.7 mmol⋅L^-1^ in prepubertal boys compared to 14.2 mmol⋅L^-1^ in adults. Similar results were also found in the current study. In fact, the peak blood lactate values were 5.76 ± 2.03 to 6.02 ± 1.58 mmol⋅L^-1^ for both CJ30s tests and 6.77 ± 2.25 mmol⋅L^-1^ for WAnT. Such results could also explain the lower glycolytic capacity and the immaturity of anaerobic metabolism in children.

As we know, anaerobic capacity represents the maximal amount of energy that can be resynthesized during a specific exercise (Green, 1994), for an isolate muscle grouping and during specific period of time (Bangsbo et al., 1990). Therefore, AC measurement is more dependent of exercise type (i.e., CJ30s vs. WAnT). AC estimated using the net blood lactate concentration and the fast component of EPOC was validated initially by Bertuzzi et al. (2010) for cycle ergometer and afterward reinforced by Miyagi et al. (2017) for cycling and from Zagatto et al. (2016a) for treadmill running, reporting similar findings to maximal accumulated oxygen deficit. In addition, this procedure was widely reliable (Zagatto et al., 2017b), sensitive to distinguish individuals with different training status (Zagatto et al., 2017b), significantly correlated with running performance (Zagatto et al., 2017a), and sensitive enough to detect alterations in glycolytic metabolism responses following buffer supplement ingestion (Brisola et al., 2015).

Hence, the lower glycolytic capacity in prepubertal children can explain the lower AC reported in this study (1.77 ± 0.88 during CJ30s and 2.05 ± 0.74 LO_2_ during WAnT) compared to adults [i.e., ∼3.6–4.0 LO_2_ (Miyagi et al., 2017)]. It is important to recognize the limitations of the immature musculoskeletal system, which is structurally different than the mature system of adult and young individuals (Boisseau and Delamarche, 2000). The maximal anaerobic power exerted by muscle on force-velocity testing is then low in children compared to adult even if it is expressed by total or lean body mass unit and especially in males (Blimkie, 1989; Dotan et al., 2012). According to Dotan et al. (2012), movement (Agonist-antagonist action) of muscle can explain this difference among different age population. As activation of antagonist muscle can reduce the maximal power developed by agonist muscle. Hence, increased agonist-antagonist co-contraction may explain the reduced maximal power in children (Dotan et al., 2012). On the other hand, numerous clinical studies demonstrated that children’s muscles are characterized by lower type-II fiber proportion compared to other age groups (Jansson, 1996), while for numerous other studies, these difference are scant (Belanger and McComas, 1989; Davis et al., 2008). These characteristics could fully or partly explain difference in anaerobic power between children and adult during high-intensity efforts (Van and Dore, 2002; Dotan et al., 2003, 2012; Nikolaidis et al., 2016a). Dotan et al. (2003) concluded that young men were 24% more powerful per unit body mass than boys, and their lactate levels were 50% greater than levels measured in younger ages. The power of the lower extremities was measured with use of the WAnT in children (Chia et al., 1997; Bencke et al., 2002; Nikolaidis et al., 2016a,c).

The WAnT was widely used for anaerobic power testing in combat sports. Ouergui et al. (2014) observed a peak power ranging 9.8 W⋅kg^-1^ and the mean power ranging to 10.3 and 6.5–7.2 W⋅kg^-1^ during WAnT in kickboxing athletes. In the current study, the peak power was 5.41 ± 1.26 W⋅kg^-1^ while the mean power corresponded to 3.92 ± 0.95 W⋅kg^-1^, which were lower than in kickboxing athletes, even in karateka’s that have the kick as a matters determinant during the fight. Nikolaidis et al. (2016b) distinguished differences in anaerobic power measurements among different age groups, with the results being more powerful in the 18–32 age groups.

High-level kickboxing performance requires high neuromuscular activation of lower limbs (Tabben et al., 2014; Nikolaidis et al., 2016b). According to Nikolaidis et al. (2016b) neuromuscular fitness including jumping ability is a main determinant of anaerobic fitness in Taekwondo athletes. Phosphagens thus act as energy-storage molecules and are especially useful during brief and short muscular activity such as actions in Karate sport. Therefore, the estimation of E_PCr_ during jump test and during WAnT is an effective method that will deliver athletes with best knowledge of their own abilities during maximal efforts.

Interestingly, the current findings showed significant correlation between E_PCr_ from CJ30s and WAnT (*r* = 0.645). This result demonstrates the possibility to estimate the phosphagen energy system using jumps. In addition, the E_PCr_ values reported in the current study (1.10 ± 0.43 and 1.33 ± 0.40 LO_2_ during CJ30s and Wingate test, respectively) are similar to those found in adult sedentary individuals (∼1.4 LO_2_), slight lowers than moderately trained runners (∼ 1.4 LO_2_), but that were estimated in treadmill running (Zagatto et al., 2017b).

Despite of importance of anaerobic metabolism in karate sport, little information concerning this topic is found in the literature, mainly about the phosphagen energy system (Doria et al., 2009; Chaabene et al., 2012; Tabben et al., 2013). In this way, when some informations are found, mainly in other combat sports, in general, are reported the capacity of mechanical power instead some information about the bioenergetical metabolism.

The current study advances a valid procedure to estimate the anaerobic capacity and anaerobic power, as well as to estimate the energetic contribution from phosphagen and glycolytic energy systems in a simple and ease effort, jumping, instead of only to measure the peak and mean mechanical power. This point is very relevant due to the fact that the anaerobic power does not represent anaerobic capacity and vice-versa (Minahan et al., 2007; Andrade et al., 2015), reinforcing the requirement of to measure the anaerobic capacity and anaerobic power separately. Therefore, we can demonstrate with these finding the possibility to use the CJ30s to measure the anaerobic power (i.e., the maximal and mean jump height of the first four jumps, the mean jump height of total jumps) and anaerobic capacity (i.e., the sum of E_PCr_ and E_[La]_).

Despite that WAnT is an effective tool for anaerobic power measurement in lower limbs, it represents insufficient tool to determine anaerobic outcomes in sports that require jumping as basic element of movement (Sands et al., 2004; Dal Pupo et al., 2014) such as Karate, therefore, it was necessary to use jumping test to assess anaerobic performance. As for WAnT, numerous type of jumping test such as vertical standard jump (VJ) and modified 30s Bosco Vertical jump test were found to have similar mechanical outcomes that expresses the anaerobic power compared with cycling tests (Theodorou et al., 2013; Dal Pupo et al., 2014; Nikolaidis et al., 2016a,c). Such findings were found to be related to age groups in study of Nikolaidis et al. (2016a), and therefore, measuring the test-reliability of jump test in younger age (≤12 years) would of great benefit in combat sport and for coaches during evaluation.

In addition, it is relevant to report that both tests have the same characteristics, to perform the maximal effort of lower limbs during 30 s, such the description of these tests (i.e., Wingate test and the modified 30 s Bosco Vertical jump test) and both anaerobic in nature given their characteristics (i.e., all-out for 30 s). However, while the Wingate test is performed during the pedaling phase in a circular movement, the CJ30s is performed during continuous jump with free joint, therefore, resulting a lower VO_2_ response during CJ30s such observed in the **Figure 1**. Nevertheless, the significant and large correlation between anaerobic capacity estimated during CJ30s and WAnT demonstrate the concurrent validity of CJ30s.

However, there are some limitations in the current study. The primary one element is the gender (female), as for this type of investigation, it would be more useful to investigate both male and female athletes. Second, blood lactate levels are not the only determinant of the glycolytic energy production and it depends on metabolic pathways of each individual (Boisseau and Delamarche, 2000) which depends on several intrinsic factors (i.e., muscle fibers recruitment) especially in young population. Therefore, caution should be taken when using these findings to analyze validity of the test. In addition, we recommend that in future study, there is a need to investigate the relationship between anaerobic capacity and power, as well as each energetic metabolism with karate performance, using the ranking performance within a player category. In fact, this information could elucidate if the anaerobic metabolism is also relevant to performance in children such it is relevant for adult athletes.

## Practical Application

The rationale behind this research argues for a need of the determination of reliability of CJ30s test to measure anaerobic performance in children by using WAnT as a reference tool. It is important to mention that CJ30s would be more suitable for Karate sport, as it involves the stretch-shortening action of lower limbs and it allows determination of anaerobic performance without using cycling movements. This test will be more adapted by athletes who do not have enough time to familiarize with cycling test. The CJ30s may be also more practical than the WAnT, as it offers easy movements with less discomfort and fatigue in children or individuals with low tolerance to exercises that require greater involvement of anaerobic pathway. In addition, in order to measure the anaerobic power and anaerobic capacity in children of combat sport, the CJ30s would be an efficient test that replaces WAnT. Finally, coaches and physical trainers could simply use the CJ30s test to measure mechanical outcomes with less economical resources.

## Conclusion

A 30 s continuous jumps test may be more practical tests compared with other longer tests (e.g., 60-s) with an improved validity and reliability. It is a specific exercise test for kicking combat sports which involve anaerobic alactic power, explosive power expressed in the stretch-shortening cycle movements. In addition, the use of a simple variable, i.e., jump height, rather than mechanical power has greater practical application and/or clinical relevance for coaches and physical trainers in combat sports. Because of its simple instrumentation, the CJ30s test is easier than other methods of anaerobic power/capacity assessment performed in the area of kicking combat sports

## Author Contributions

All authors listed have made a substantial, direct, and intellectual contribution to the work, and approved it for publication.

## Conflict of Interest Statement

The authors declare that the research was conducted in the absence of any commercial or financial relationships that could be construed as a potential conflict of interest. The reviewer PN declared a past co-authorship with one of the authors to the handling Editor. The reviewer JB-G and handling Editor declared their shared affiliation.
